# Toward a new ‘EPOCH’: optimising treatment outcomes with phosphodiesterase type 5 inhibitors for erectile dysfunction

**DOI:** 10.1111/j.1742-1241.2009.02119.x

**Published:** 2009-08

**Authors:** R Sadovsky, G B Brock, S W Gutkin, S Sorsaburu

**Affiliations:** 1 State University of New York, Downstate Medical CenterBrooklyn, NY, USA; 2 Division of Urology, Faculty of Medicine and Dentistry, University of Western OntarioLondon, ON, Canada; 3 Rete Biomedical Communications Corp.Wyckoff, NJ, USA; 4 Eli Lilly and CompanyIndianapolis, IN, USA

## Abstract

Despite the marked adverse impacts of erectile dysfunction (ED) on quality of life and well-being, many patients (and/or their partners) do not seek medical attention for this problem, do not receive treatment or discontinue such treatment even when it has effectively restored erectile responses to sexual stimulation. Phosphodiesterase type 5 (PDE5) inhibitors are considered first-line therapies for men with ED. To help physicians maximise the likelihood of treatment success with these agents, we conducted an English-language PubMed search of articles involving approved PDE5 inhibitors dating from 1 January 1998 (the year in which sildenafil citrate was introduced), through 31 August 2008. In addition to sildenafil, tadalafil and vardenafil, search terms included ‘adhere*’, ‘couple*’, ‘effect*’, ‘effic*’, ‘partner*’, ‘satisf*’, ‘succe*’ and ‘treatment outcome.’ Based on our analysis, physician activities to promote favourable treatment outcomes may be captured under the mnemonic ‘EPOCH’: (i) Evaluating and educating patients and partners to ensure realistic expectations of therapy; (ii) Prescribing a treatment individualised to the couple’s lifestyle needs and other preferences; (iii) Optimising treatment outcomes by scheduling follow-up visits with the patient to ‘fine-tune’ dosages and revisit key educational messages; (iv) Controlling comorbidities via lifestyle counselling, medications and/or referrals and (v) Helping patients and their partners to meet their health and psychosocial needs, potentially referring them to a specialist for other forms of therapy if they are not satisfied with PDE5 inhibitors.

**Review Criteria**

A PubMed search of the English-language literature was conducted covering the period of 1 January 1998 [the year that the PDE5 inhibitor sildenafil citrate (Viagra®; Pfizer) was approved for use in many markets], through 31 August 2008. In addition to sildenafil, tadalafil and vardenafil, search terms included ‘adhere*’, ‘couple*’, ‘effect*’, ‘effic*’, ‘partner*’, ‘satisf*’, ‘succe*’ and ‘treatment outcome’.

**Message for the Clinic**

Erectile dysfunction (ED) can adversely affect quality of life in men and their sexual partners. Despite > 10 years of experience with PDE5 inhibitors, many couples (∼30%) experience suboptimal treatment outcomes and discontinue therapy. Physicians can improve ED treatment outcomes by effectively Evaluating and educating patients and/or their partners; Prescribing and Optimising PDE5 inhibitor regimens; Controlling comorbidities that can undermine responses and/or Helping couples to identify an alternative therapy.

## Introduction

In the 1992 National Institutes of Health (NIH) Consensus Development Conference, impotence was defined as ‘inability of the male to attain and maintain erection of the penis sufficient to permit satisfactory sexual intercourse’ (1). There are two relevant aspects of this definition. First, ‘satisfactory sexual intercourse’ usually includes participation of a partner, thus rendering the problem a couple-based condition (2). Second, NIH panellists included the patient-related outcome of satisfaction in the definition of erectile dysfunction (ED) as a clinical entity. Satisfactory ED treatment outcomes are subjective, span psychosocial and medical domains, and are hence determined chiefly by the patient and his partner in consultation with a physician.

For many patients and their sexual partners, ED reduces quality of life (QOL) and causes emotional distress (3–9). However, despite an emerging candour about ED and widespread educational activities supporting phosphodiesterase type 5 (PDE5) inhibitors since approval of sildenafil citrate in many markets (in 1998), these medications (and ED in general) continue to be misunderstood and/or ineffectively used. As a result, there are perhaps millions of men experiencing the ‘insult’ of ineffective PDE5 inhibitor treatment added to the ‘injury’ of ED. Up to 52% of US middle-aged and older men have ED (10–12); however, many do not seek medical attention (13), do not receive prescription treatment [84–93% (10,13)] or discontinue treatment (10,13–15).

In fact, one in three men discontinue ‘successful’ treatment with a PDE5 inhibitor [i.e. treatment that restores erectile function (EF)], sometimes after the first prescription (16,17). In one study, 54 (35%) of 156 patients with successful restoration of normal EF using sildenafil discontinued treatment after 6 months. Reasons included patients and/or partners not being emotionally ready to resume sexuality after a long abstinence (37%); concerns about medication adverse effects (18%); return of spontaneous erections (15%); unwillingness to accept a ‘drug-dependent erection’ (7%) and either the unacceptability of planned sexual activity or lack of sexual interest (4% each) (17).

In light of these considerable challenges, the aim of this review was to examine practical strategies that primary-care physicians and others can adopt to help optimise treatment outcomes once a patient or couple has decided to receive therapy with a PDE5 inhibitor; and increase overall ‘therapeutic yield’, or the numbers of patients experiencing optimal outcomes.

## Methods

### Data sources and extraction

A PubMed search of the English-language literature was conducted covering the period of 1 January 1998 [the year that the PDE5 inhibitor sildenafil citrate (Viagra®; Pfizer, New York, NY, USA) was approved for use in many markets], through 31 August 2008.

Our search used the following terms: (i) title words ‘phosphodiesterase’, ‘PDE5’, ‘sildenafil’, ‘tadalafil’ (Cialis®; Eli Lilly, Indianapolis, IN, USA) and ‘vardenafil’ [vardenafil hydrochloride (Levitra®); Bayer, Wayne, NJ, USA], each separated by the Boolean operator ‘OR’; (ii) NIH National Library of Medicine medical subject headings (MeSH terms) ‘human’, ‘impotence’ and ‘treatment outcome’ (each separated by OR); (iii) title terms ‘adher*’, ‘choice*’, ‘compl*’, ‘couple*’, ‘discont*’, ‘effect*’, ‘effic*’, ‘fail*’, ‘optim*’, ‘partner*’, ‘prefer*’, ‘quality’, ‘respon*’, ‘satisf*’, ‘sexual*’ and ‘succe*’ (each separated by OR); (iv) the Boolean operator ‘NOT’ and publication types ‘letter’, ‘editorial’ or ‘review’ and (v) the operator NOT and ‘benign’, ‘BPH’, ‘pulmonary’, ‘PAH’, ‘PPH’ and ‘PH’ (abbreviations refer to benign prostatic hyperplasia, pulmonary arterial hypertension, primary pulmonary hypertension and pulmonary hypertension, respectively) because these latter terms refer to potential non-ED indications. The search produced 182 citations, 93 of which were included. Pilot studies were excluded, as were trials conducted outside North America, consistent with the English-language reference search and to limit the scope of the review. Also excluded were studies involving second-line PDE5 inhibitors, diagnostics and other special clinical settings, and investigations involving unapproved medications or off-label uses of medications. To complement this search and to help survey other associated therapies in patients with suboptimal responses to PDE5 inhibitors, a second search was conducted, which substituted the following title terms in (iii) above: ‘androg*’, ‘cardio*’, ‘coron*’, ‘counsel*’, ‘horm*’, ‘life*’, ‘mood’, ‘obes*’, ‘overweight’, ‘prost*’ and ‘testos*’. This search produced 12 additional citations, 10 of which were included; the other two involved a pilot study and a clinical trial concerning second-line sildenafil use after intracavernosal injection therapy (ICIT).

These literature searches were supplemented by reviewing manufacturer labelling for each PDE5 inhibitor (18–20) and by including other relevant articles, including consensus treatment guidelines, as well as by reviewing article bibliographies.

## Results

**Figure 1:**
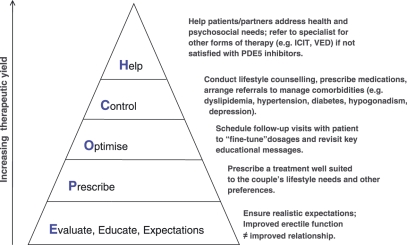
Physician activities to optimise treatment of erectile dysfunction with phosphodiesterase type 5 (PDE5) inhibitors can be readily remembered by the mnemonic ‘EPOCH’. ICIT, intracorporeal (intracavernosal) injection therapy; VED, vacuum erection device

According to our data review, physician activities to help maximise the likelihood of satisfactory treatment outcomes and promote medication adherence with PDE5 inhibitors can be subsumed under the mnemonic ‘EPOCH’: E = evaluate/educate/expectations, P = prescribe, O = optimise/titrate, C = control comorbidities/counsel, H = help the patient and his partner identify (non-PDE5-based) satisfactory therapy (Figure 1).

### Evaluate/educate/expectations

#### Evaluate

**Figure 2:**
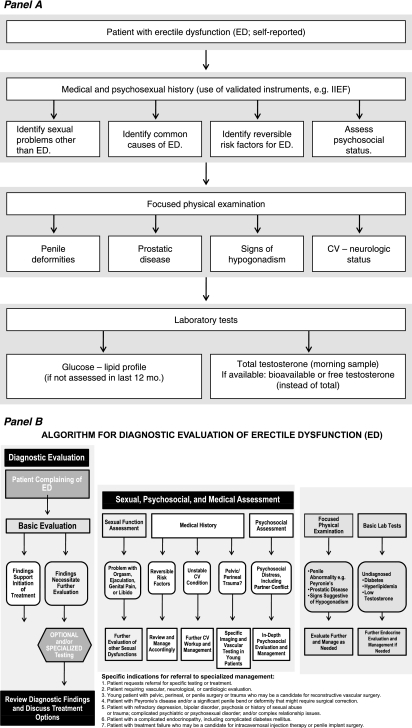
Evaluation of the patient with erectile dysfunction (ED) by consensus guidelines. Panel (A) Minimal diagnostic evaluation (basic workup) according to the European Association of Urology (21). Panel (B) Complete diagnostic algorithm for ED according to the World Health Organization and other health authorities (22), including guidelines for referrals of patients to specialists. CV, cardiovascular; IIEF, International Index of Erectile Function. Reproduced with permission from Wespes et al. (21) and Lue et al. (22). Panel A reprinted from EAU guidelines on erectile dysfunction: an update. Vol 49, Wespes E, Amar E, Hatzichristou D et al., 806–815, 2006, with permission from Elsevier. Panel B reproduced with permission of Blackwell Publishing Ltd., Summary of the recommendations on sexual dysfunctions in men, Lue TF, Giuliano F, Montorsi F et al. 2004

Consensus guidelines recommend, at minimum, a medical, sexual and psychosocial history; and a focused physical examination and laboratory tests (Figure 2A; 21,22). A complete medical history places the diagnosis of ED in the appropriate medical context for each patient. A physical examination of the genitourinary system can identify anatomic causes of ED, which might warrant referral to a urologist (23,24). A more comprehensive workup, including recommendations for referrals, is shown in Figure 2(B) (22). The clinician can also use the International Index of Erectile Function (25) or the Sexual Health Inventory for Men (SHIM) (26), which involves (i) erection confidence, (ii) erection firmness, (iii) frequency of maintaining erection, (iv) frequency of maintaining erection to completion of intercourse and (v) intercourse satisfaction.

Erectile dysfunction may be viewed as a ‘portal to men’s health’ (27). A diagnostic workup for comorbidities may assist not only in optimising treatment but also in identifying other conditions, including depressive disorders, male hypogonadism and the presence of other cardiovascular risk factors. Endothelial dysfunction represents the ‘common denominator’ between ED and cardiovascular disease (CVD) (28). The presence of ED confers a 1.45-fold increased risk of CVD (29,30).

The recommendations of the Second Princeton Consensus Conference provide useful diagnostic and management algorithms for patients with ED and CVD (31–33). Most men with such conditions who are not taking nitrates may be treated with PDE5 inhibitors. Obtaining a complete medical history is also important to uncover potentially modifiable iatrogenic causes of ED (Table 1; 34).

**Table 1:** Evaluation: potential iatrogenic causes of sexual dysfunction, including erectile dysfunction

Cause	Effect	Probable mechanism
**Medication**		
Antidepressants	ED, ↓sexual desire, retarded ejaculation	Descending inhibitory input to sacral serotonergic ‘sex center’ + peripheral anticholinergic effects; potentially reversed by cyproheptadine (serotonin antagonist) or bethanechol (cholinergic)
Antihypertensives (e.g. α-methyldopa, reserpine)	↓Sexual desire, ED, retarded ejaculation	Deplete central dopamine, which mediates neural input related to sexuality (in hypothalamus/paraventricular nucleus); lower blood pressure; direct actions in corpus cavernosum (e.g. intracellular calcium regulation); effects on neurotransmitters/hormones (e.g. ↑prolactin)
Other cardiovascular agents (e.g. digoxin, disopyramide, antihyperlipidemics, propranolol)	ED, ↓sexual desire	Digoxin ↓testosterone and ↑oestrogen levels because of similar structure to sex steroids; digoxin also blocks Na-K-ATPase pump with net increase in intracellular calcium and increased corporeal smooth-muscle tone (anti-erectile effects)
Diuretics	ED (thiazide), ↓sexual desire, gynaecomastia and/or mastodynia (spironolactone, bendrofluazide, HCTZ)	Spironolactone blocks testosterone synthesis and competitively binds to androgen receptors
Antipsychotics (neuroleptics)	↓Sexual desire, ED, retarded ejaculation (with or without priapism)	Block pituitary/hypothalamic dopamine receptors; ↑prolactin levels; anticholinergic activity + α-adrenergic activity; indirect effects secondary to weight gain, CNS sedation, parkinsonism, psychomotor retardation
Drugs of abuse (e.g. cocaine, amphetamines)	ED	Diffuse atherosclerotic changes/endothelial toxicity + ↑α-adrenergic activity on chronic use
Histamine (H_2_) blockers	↓Sexual desire (cimetidine), ED, gynaecomastia	Anti-androgen activities/blockade of androgen receptors; ↑prolactin; direct corporeal effects
Anti-androgens (e.g. finasteride)	ED, reduced sexual desire	Block androgen synthesis; oestrogen, ketoconazole and digoxin may lower serum testosterone and/or competitively bind to androgen receptors
Surgery/radiation/brachytherapy (e.g. for prostate cancer)	ED	Effects on neurovascular structures

Adapted from Rehman and Melman (34) with permission. ED, erectile dysfunction; HCTZ, hydrochlorothiazide; CNS, central nervous system. Adapted from (CMG and Atlas of Clinical Urology: Impotence and Infertility. Vol 1. Philadelphia, PA: Current Medicine Inc; 1999:1.1–1.16, Figure 1–17, page 1.14, Rehman J, Melman A. Pathophysiology of erectile dysfunction.) with kind permission of Current Medicine Group, LLC. © 1999 All rights reserved

#### Educate

Counselling patients about ED treatments need not be time consuming and can enhance treatment. Recent studies found that weekly group counselling sessions plus sildenafil treatment were associated with superior responses on the SHIM compared with sildenafil alone or counselling alone (35–37). In a study involving primary-care physicians spending about 12 min per patient, approximately 42% of patients who had failed to respond to sildenafil and were eligible for analysis achieved salvage after being re-educated (38). Salvage, defined by positive responses to the global assessment questionnaire after initially not showing such responses, has also been achieved in patients using daily tadalafil after suboptimal responses to on-demand therapy (39). At minimum, a few central educational messages concerning PDE5 inhibitors should be communicated (Table 2; 40–45).

**Table 2:** Education and expectations: key educational messages, common myths and cognitive distortions in men with erectile dysfunction

**Key educational messages**
PDE5 inhibitors are not ‘erectogenic’ agents *per se*. They are considered ‘contingent agonists’ of the sexual response and are hence effective only in the presence of sexual arousal/stimulation
Sufficient arousal and sexual stimulation are particularly important in elderly men, including those with diabetes, who may have increased sensory (tactile) thresholds (41,42)
PDE5 inhibitors are not always successful in restoring erectile function adequate for sexual intercourse on the first attempt. Each PDE5 inhibitor should be taken ≥ 4 times before it is deemed ineffective
**Myths**
It is the responsibility of the man to satisfy the woman
Size and firmness of the erect penis are necessary determinants of the female partner’s satisfaction
A woman’s favourite part of sex is intercourse
A man always wants and is always ready to have sex
With age, all men lose their ability to achieve erections
**Cognitive distortions**
All-or-nothing thinking, e.g. ‘I am a complete failure because my erection was not 100% rigid’
Overgeneralisation, e.g. ‘If I had trouble getting an erection last night, I won’t have one this morning’
Disqualifying the positive, e.g. ‘My partner says I have a good erection because she doesn’t want to hurt my feelings’
Mind reading, e.g. ‘I don’t need to ask. I know how she felt about last night’
Fortune telling, e.g. ‘I am sure things will go badly tonight’
Emotional reasoning, e.g. ‘Because a man feels something is true, it must be’
Categorical imperatives, e.g. ‘should’, ‘ought to’ and ‘must’ dominate the man’s cognitive processes
Catastrophising, e.g. ‘If I fail to achieve an erection tonight, my partner will abandon me’

Adapted with permission from Althof and Wieder (45). Adapted with kind permission from Springer Science+Business Media: Endocrine, Psychotherapy for erectile dysfunction: now more relevant than ever. Vol 23, 2004, page 132, Althof SE, Wieder M, Section IV. Permission also obtained from Althof SE

Pharmacokinetic differences among the three PDE5 inhibitors may enable the physician to individualise treatment. Tadalafil has a longer half-life (17.5 h) than sildenafil (4–5 h) or vardenafil (4–5 h), promoting erectile responses to arousal for up to 36 h after dosing (46). The half-life of sildenafil promotes erectile responses to arousal for up to 12 h (47), whereas the half-life of vardenafil does so for up to 8 h (48). Certain couples may appreciate having more ‘spontaneous’, ‘relaxed’ or ‘natural’ sex with less planning around dosing. Studies have demonstrated patient and/or partner preferences for and higher satisfaction with tadalafil compared with other PDE5 inhibitors, but many of these studies had design limitations (49–59). On the other hand, many couples preferring sildenafil may value the fact that it has the longest postmarketing surveillance record (10 years), while those preferring either sildenafil or vardenafil may value the more rapid reversibility of these agents' pharmacodynamic effects.

Despite certain differences in PDE5 inhibitors, clinical trials suggest that monotherapy with either sildenafil, tadalafil or vardenafil restores erectile responses to sexual arousal in most patients, including those seen in different treatment settings, those of different ages and ethnicities, and/or those with ED of varying severities, aetiologies, comorbidities and other conditions (46,60–81). Such treatment may also improve sexual satisfaction, self-esteem, QOL, depressive symptoms, communication and other psychosocial outcomes for patients and/or their partners (3,49–56,60–64,82–84). All three agents potentiate erectile response and permit successful intercourse in most men within 30–60 min after dosing on an empty stomach (46,65–68). Data have shown that the duration of erection sufficient for successful sexual intercourse was more than double (12.8 ± 1.00 min) in men taking vardenafil 10 mg 60 min before intercourse compared with that of counterparts receiving placebo (5.4 ± 1.00 min; p < 0.001) (85,86).

#### Expectations

**Figure 3:**
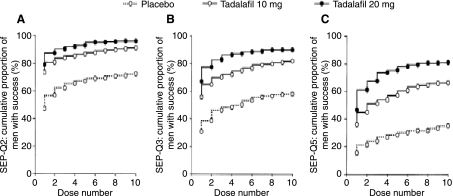
Cumulative proportion of men taking tadalafil able to achieve (A) their first successful penetration [Sexual Encounter Profile Question (SEPQ) 2], (B) first successful intercourse (SEPQ3) and (C) to experience sexual satisfaction (SEPQ5) by dose. Reprinted from Schulman et al. (43). Reprinted from Urology, 64, Schulman CC, Shen W, Stothard DR, Schmitt H, Integrated analysis examining first-dose success, success by dose, and maintenance of success among men taking tadalafil for erectile dysfunction, 783–8, 2004, with permission from Elsevier.

Researchers have conducted analyses to determine factors influencing QOL, mood and sexual satisfaction in patients and their partners (87). Defining satisfaction relates to the degree to which treatment outcomes are compatible with expectations and it is considered instrumental in maintaining long-term treatment adherence with PDE5 inhibitors (40). To help promote treatment satisfaction, it is useful to identify any false expectations about sexual function and the effects of PDE5 inhibitors. Patients and partners should not expect successful intercourse on the first attempt after the first use of a PDE5 inhibitor (Figure 3; 43) [Each PDE5 inhibitor should be taken ≥ 4 times before it is deemed ineffective (Table 2; 21)]. Althof and Wieder (45) have identified some other key myths to debunk (Table 2). Treatment with PDE5 inhibitors also does not ensure more satisfying sex or a more satisfying relationship.

### Special case for evaluation, education and expectations: importance of the sexual partner

Patients and partners may have different needs, expectations or priorities for ED treatment, which may vary depending on religion, culture, patient age and other factors (88). It has been written that ‘when…sexual inadequacy is present, the couple is the patient’ (89). The interdependence of sexual function/satisfaction between men with ED and their partners has been demonstrated by Heiman et al. (84), among other investigators. The overall health, sexual and emotional status of the female partner can exacerbate sexual dysfunction in the ED patient. In one survey of female partners of men with ED, 82% rated sexual activity as an important aspect of life, 76% reported being sexually active and 40% reported engaging in vaginal intercourse, yet 34% reported sexual dissatisfaction, 30% arousal/lubrication difficulty, 26% anxiety/inhibition, 24% orgasmic difficulty, 18% dyspareunia and 8% incontinence during intercourse. A total of 44% had depressive symptoms (90).

In the Female Experience of Men’s Attitudes to Life Events and Sexuality (FEMALES) study, partners of men with ED reported significant deficits in sexual desire, arousal, orgasm and overall sexual satisfaction, with the degree being significantly correlated with ED severity (91). Conversely, PDE5 inhibitor therapy in the male patient was associated with improved sexual experiences in the partner, and was particularly effective in men with severe ED and a supportive female partner (91). Similar findings have been reported in trials involving each of the PDE5 inhibitors (54,63,92).

Although the partner should be involved in ED assessment and treatment planning as early as possible (22), consensus guidelines have stated that no patient with ED should be denied treatment solely on the basis of his partner’s being unwilling or unable to accompany him to the initial ED office visit (93). Whether the partner decides to accompany the patient is largely informed by sociocultural factors and individual preferences and needs (22). When the partner is not available, it is still important to ask about her sexual health and satisfaction, and it may ultimately be necessary to address her sexual problems to optimise ED treatment outcomes (30,89). According to Althof, ‘before concluding that treatment has failed, the clinician must consider the myriad biopsychosocial reasons that could…prevent the couple from attaining their stated goal’ (94). Such ‘biopsychosocial obstacles’ include a long period of sexual abstinence before treatment, a suboptimal non-sexual relationship and/or the female partner not being emotionally and/or physically prepared to resume lovemaking (94). Other potential issues in the female partner include an overall aversion to sex, sexual pain (e.g. vaginismus, dyspareunia) and low desire/arousal (30). It is often useful to invite the patient and partner to the office in the event of PDE5 inhibitor treatment failure, to better appreciate the sources of problems.

Successful ED treatment should accommodate the behavioural complexity of sexual intimacy, including patient–partner communication. Predisposing, precipitating and maintaining factors, as well as the presence and quality of morning erections and partner issues related to sexual aversion, menopause, vaginal atrophy/pain or dyspareunia, should be assessed (Table 3; 30). Women need to be physically and emotionally ready to resume or increase their amount of lovemaking with the patient. For example, treatment recommendations (including the use of local oestradiol) have been developed for sexual pain associated with vaginal atrophy, which is very common in women > 50 years of age (30,92,95,96) and may also result from diabetes or as a side effect of certain medications.

**Table 3:** Causes of erectile dysfunction

Predisposing	Precipitating	Maintaining
Lack of sexual knowledge	New relationship	Relationship problems
Poor past sexual experience	Acute relationship problems	Poor communication between partners
Relationship problems	Family/social pressures	Lack of knowledge about treatment options
Religious/cultural beliefs	Pregnancy/childbirth	Ongoing physical or mental health problems
Restrictive upbringing	Other major life events	Other sexual problems in the man or his partner
Unclear sex/gender preference	Partner’s menopause	Drugs (see also Table 1)
Previous sexual abuse	Acute physical or mental health problems	
Physical/mental health problems	Lack of knowledge about normal changes of ageing	
Other sexual problems in the man or his partner	Other sexual problems in the man or his partner	
Drugs (see also Table 1)	Drugs (see also Table 1)	

Adapted with permission from Hackett et al. (30). Adapted with permission of Blackwell Publishing Ltd., Hackett G. Kell P, Ralph D et al, British Society for Sexual Medicine guidelines on the management of erectile dysfunction. J Sex Med 2008; 5: 1841–65.

Female sexuality is complex; research concerning the pathophysiology of, and potential pharmacotherapy for, female sexual dysfunction lags significantly behind that concerning male sexual dysfunction (97,98). In this context, it has been asserted that ‘despite recognising the importance of women’s sexual health, few healthcare providers have the knowledge to treat women, or men and women together as a couple’ (98).

These limitations aside, female partners with sexual dysfunction should be treated where possible, provided with sound education or referred to specialists as appropriate. According to recent consensus guidelines, ‘As sex is a subjective experience…all couples affected by sexual dysfunction have…some psychological component to their problem. Almost all…will benefit from simple sex education’ (30). This includes ‘behavioural advice regarding foreplay, sexual activity and the integration of medication into the couple’s sexual behaviour’ (30). When the psychosocial history or other discussions with the patient and partner identify psychological and relationship issues, couples may benefit from psychosexual or couples therapy. Sex therapists can help to instruct patients and partners in sexual enhancement techniques and also to address relationship problems. In many cases, sexual problems represent manifestations of broader communication and relationship issues, including mistrust, anger and power struggles (99). Formal cognitive-behavioural interventions by professional therapists may also benefit some couples (30).

Referrals to sex therapists can be arranged through the American Association of Sexuality Educators Counselors and Therapists (Ashland, VA; http://www.aasect.org; 804-752-0026) and to marital therapists through the American Association for Marriage and Family Therapy (Alexandria, VA; http://www.aamft.org or http://www.therapistlocator.net/; 703-838-9808).

In summary, the primary-care physician is ideally poised to help patients with ED and their partners by assessing the problem; providing sound education, including a focus on pleasure and arousal (via more prolonged foreplay and sensuality) rather than erection; and/or referring couples to a sex therapist, psychotherapist or marriage counsellor, as needed (99). Finally, medical therapy and counselling couples or psychosexual therapy are not mutually exclusive; recent evidence suggests that a combination of such therapies can enhance sexual satisfaction, intimacy and cognition in men with ED and their female partners (100).

### Prescribe

#### Available regimens

Phosphodiesterase type 5 inhibitors are taken ‘on demand’ or ‘as needed’ before sexual activity, up to once daily. Vardenafil is available in four strengths (2.5, 5, 10 and 20 mg), allowing for dosing flexibility. Sildenafil is available in three strengths (25, 50 and 100 mg). Tadalafil is the only PDE5 inhibitor approved for once-daily dosing (as 2.5- and 5-mg tablets) and on-demand dosing (5, 10 and 20 mg). On-demand dosing may be ideal for some couples, whereas others may benefit from a low-dose daily dosing regimen that does not require co-ordination of sexual activity and may particularly benefit men wishing to have sex at least twice weekly. A recent publication suggested that efficacy and tolerability profiles are similar when tadalafil (2.5 or 5 mg) is administered once daily compared with as needed, and suggested a favourable risk-benefit balance for once-daily treatment (69).

#### Pharmacodynamics

Phosphodiesterase type 5 inhibitors potentiate erectile responses to sexual arousal by blocking the enzymatic degradation (by PDE5) of cyclic guanosine monophosphate. They are highly selective for PDE5, with an *in vitro* potency ratio of 41 :2 : 1 for vardenafil:tadalafil:sildenafil (70).

Many adverse events encountered by men taking PDE5 inhibitors, including dyspepsia, headache, nasal congestion and back pain, probably relate to vasodilation in non-corporeal (non-penile) vascular beds. Much less frequently, certain visual adverse events have been reported, including non-arteritic anterior ischaemic optic neuropathy (101–107), although their causal relationship to treatment is unclear. Sudden sensorineural hearing loss has recently been added to the prescribing information of all three PDE5 inhibitors (18–20).

#### Pharmacokinetics

All three PDE5 inhibitors are absorbed rapidly after oral dosing, reaching maximum plasma concentrations approximately 1 h after dosing for sildenafil and vardenafil and 2 h after dosing for tadalafil. High-fat meal intake can influence the absorption of sildenafil and vardenafil, whereas tadalafil can be taken without regard to meal intake or ‘intrinsic’ factors such as age and diabetes mellitus (18–20,71–73). Low starting doses are recommended for elderly men taking sildenafil or vardenafil; tadalafil dosage adjustment is not necessary for otherwise healthy elderly men (18–20). Patients are instructed to take PDE5 inhibitors approximately 1 h before sexual activity, up to once daily. Zinner (74) reported no significant decline in efficacy when sildenafil was taken 1 h before or during a meal, and approximately 82% of men reported successful intercourse > 10 h after sildenafil dosing.

All three PDE5 inhibitors are distributed widely throughout tissues and are extensively (≥ 94%) protein bound, with foecal excretion as the main route of elimination. They are also substrates for oxidative metabolism by cytochrome P450 3A4/5 (CYP3A4/5) isoenzymes. Drug-drug interactions that can compromise efficacy include concomitant PDE5 inhibitors and CYP3A4/5 inducers, such as rifampin. Interactions that can compromise tolerability include concomitant PDE5 inhibitors and CYP3A4/5 inhibitors. Reduced doses of PDE5 inhibitors are recommended when they are co-administered with certain human immunodeficiency virus protease inhibitors as well as other CYP inhibitors.

### Optimise/titrate

Follow-up visits are important for optimising patient and partner responses to ED treatment. The first step is to educate men concerning the salient pharmacological properties of each PDE5 inhibitor that may affect usage patterns and treatment outcomes. With sildenafil, this has often involved educating patients about the availability of the 100-mg dose to optimise erectile hardness and other intermediate outcomes (75–77). A study of 100 consecutive sildenafil non-responders showed that 45% of patients had never taken the highest dose (100 mg), 32% took sildenafil after eating, 22% took the medication immediately before initiation of sexual activity rather than 1 h before and 12% were unaware that sexual stimulation was necessary to achieve an erection (78). Only approximately one in three men reported that their physicians had scheduled follow-up visits. After addressing some of these issues, 31% of prior non-responders achieved adequate responses to sildenafil.

**Figure 4:**
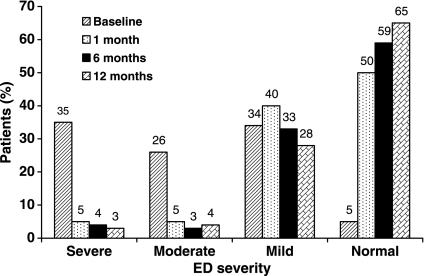
Severity of erectile dysfunction by the International Index of Erectile Function (Erectile Function domain) at baseline and tadalafil treatment months 1, 6 and 12 among men continuing to take tadalafil (10–20 mg) at 12 months (*n* = 1319). Reproduced from Roumeguère et al. (109). Reproduced with permission of Blackwell Publishing Ltd., Roumeguère T, Verheyden B, Arver S et al. Therapeutic response after first month of tadalafil treatment predicts 12 months treatment continuation in patients with erectile dysfunction: results from the DETECT study. © 2008 International Society for Sexual Medicine

Similarly, patients taking tadalafil attempted intercourse up to 36 h after tadalafil dosing when instructed that they could do so (79–81,108). Data from the recent, 12-month DETErminants of Continued use of Tadalafil (DETECT) study suggested that early success with tadalafil was a determinant of long-term outcomes, including medication adherence (Figure 4; 109).

**Figure 5:**
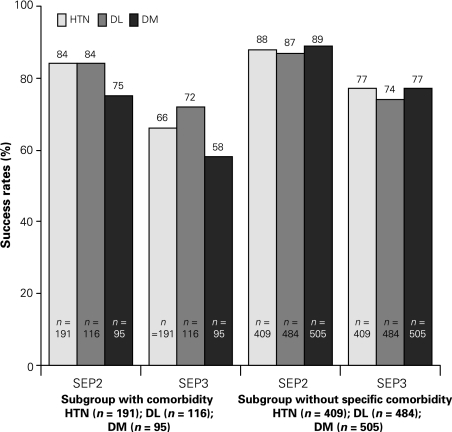
First-dose success in achieving vaginal penetration (SEP2) and intercourse completion (SEP3) in men during an open-label vardenafil challenge phase. Patients included men with comorbidities, such as diabetes mellitus (DM), dyslipidaemia (DL) or hypertension (HTN). Reproduced from Valiquette et al. (114). Reproduced from Valiquette et al. (114) © 2008 with permission from Canadian Urological Association

Researchers have evaluated first-dose successful responses to PDE5 inhibitors as a study end-point (110–113). In the recent Reliability of Vardenafil for Erectile Dysfunction (RELY)-II trial, 70–81% first-dose treatment success was reported with high-dose (20 mg), on-demand vardenafil therapy in patients with numerous comorbidities (Figure 5; 114). Maximising first-dose success with PDE5 inhibitors must be balanced against the risk of suboptimal tolerability of higher doses.

In a prospective, open-label, flexible-dose study involving sildenafil, Steidle et al. (75) identified patient comments as to dissatisfaction with sildenafil treatment, as well as steps that physicians can take to enhance treatment outcomes (Table 4).

**Table 4:** Possible reasons why patients were not completely satisfied (and possible personalised instruction to improve efficacy and satisfaction) with sildenafil

Patient comment	Possible clinical response
Treatment could work faster	Take the tablet on an empty stomach (i.e. either before a meal or ≥ 2 h after a meal); increase sexual stimulation (e.g. foreplay, caressing)
Treatment could last longer	If at 50-mg dose, increase the dose to 100 mg; increase sexual stimulation (e.g. foreplay, caressing); ensure that the subject is not waiting too long after taking sildenafil before attempting intercourse
Erections were not always completely hard	If at 50-mg dose, increase the dose to 100 mg; increase sexual stimulation (e.g. foreplay, caressing); take on an empty stomach; ensure that the subject is not attempting intercourse too soon after taking sildenafil
Patient wanted erection to last long enough to complete intercourse and ejaculation	If at 50-mg dose, increase the dose to 100 mg; increase sexual stimulation (e.g. foreplay, caressing); take on an empty stomach; try having sexual activity 30 min to 1 h after taking the medication; determine if subject is experiencing other sexual dysfunction, such as delayed ejaculation or anorgasmia, and treat accordingly
Treatment sometimes did not work perfectly	If at 50-mg dose, increase the dose to 100 mg; increase sexual stimulation (e.g. foreplay, caressing); take on an empty stomach; ensure that the subject is not waiting too long or trying too soon after taking the medication to attempt intercourse
Patient wanted to have sex more than once with the same dose of sildenafil	If at 50-mg dose, increase the dose to 100 mg; increase sexual stimulation (e.g. foreplay, caressing); take on an empty stomach; ensure that the subject is not waiting too long after taking the medication to attempt intercourse the second time
Patient experienced side effects	Remind patients that most side effects are transient, lasting a few minutes to an hour or so Headache: take appropriate treatment before taking study medication; consider decreasing dose Dyspepsia: take appropriate treatment before taking study medication; consider decreasing dose Flushing: consider decreasing dose Nasal congestion: take appropriate treatment before taking study medication; consider decreasing dose

Reproduced from Steidle et al. (75). Reprinted by permission from Macmillan Publishers Ltd: Int J of Impot Res (19) 2007.

### Control comorbidities/counsel

To our knowledge, limited high-quality, randomised, controlled trials have compared PDE5 inhibitor treatment outcomes in patients with or without adequate control of comorbidities, including serum lipids, haemoglobin A1c, blood pressure, endocrine levels/function and/or psychiatric difficulties. However, these conditions can compromise EF and warrant consideration, particularly when initial responses to PDE5 inhibitors are suboptimal.

#### Male hypogonadism

There is a substantial frequency of comorbid hypogonadism with ED, and the conditions also often coexist with major depressive disorder, cardiovascular disease, diabetes and/or metabolic syndrome; however, male endocrine decline is not inevitable (115–119). Age-related declines in reproductive hormone output in men may be accompanied by sexual dysfunction, as well as reduced lean muscle mass, bone density and sense of well-being.

Because hypogonadism can attenuate male sexual desire, some patients with suboptimal responses to PDE5 inhibitors may benefit from adjunctive exogenous testosterone treatment. Adjunctive transdermal testosterone and oral testosterone undecanoate have been evaluated in combination with sildenafil (120–126). In randomised, double-blind, placebo-controlled trials, T-gel enhanced sexual function, including nocturnal erections, circulating testosterone level and intercourse frequency, in hypogonadal men with initially suboptimal responses to sildenafil (120). To our knowledge, correlation between ‘restorative’ testosterone levels and improved EF was found in only one reported trial (126).

Safety issues surrounding testosterone treatment were reviewed in the *New England Journal of Medicine* in 2004 (127,128) and, more recently, Miner et al. developed best-practices guidelines (129). These included obtaining a serum testosterone level when an adult man shows symptoms or signs of hypogonadism and as a component of routine screening beginning at 40–50 years of age. Among symptomatic men with a serum testosterone level < 300 ng/ml, testosterone replacement therapy may be considered. Effective treatment with a PDE5 inhibitor may alter the testosterone:oestradiol ratio and play a role in moderating EF (130).

#### Cardiovascular lifestyle factors and counselling

Behavioural factors with potential adverse vascular effects can also adversely affect EF. Tobacco use is associated with vasoconstriction, endothelial toxicity, reduced penile blood flow and reduced corporeal smooth-muscle relaxation. In a multivariate analysis, only current smoking, hypogonadism and baseline ED severity significantly predicted PDE5 inhibitor treatment failure (131).

In a study involving 110 obese men [body mass index (BMI) ≥ 30 kg/m^2^], those who lost ≥ 10% total body weight had significant improvements from baseline in EF compared with controls. On multivariate analysis, changes in BMI, body weight and the waist:hip ratio were significant independent factors for improved EF (132). Moderate (∼13%) weight loss in obese men and women has also been significantly associated with improvement in sexual QOL (133).

Erectile function may be supported by medical management of comorbid cardiovascular conditions. A German group recently reported the treatment of > 1000 consecutive patients with metabolic syndrome using an angiotensin II receptor blocker (ARB) with or without hydrochlorothiazide (HCTZ) for 6 months (134). The frequency of ED declined from 78.5% at baseline to 63.7% at treatment month 6, and men receiving an ARB with or without HCTZ experienced significant improvements in EF, orgasmic function and intercourse satisfaction. Treatment with PDE5 inhibitors in men with hypertension, including those receiving concomitant antihypertensive medications [diuretics, β-blockers and angiotensin-converting enzyme (ACE) inhibitors], has been reported to be effective and well-tolerated in managing ED (135–138).

In a small, placebo-controlled, double-blind trial, adjunctive treatment with an ACE inhibitor (+sildenafil) significantly augmented EF and reduced ED symptoms compared with placebo in men with suboptimal responses to the PDE5 inhibitor (p < 0.01), whereas treatment with a statin also numerically improved EF (139). In another, smaller trial, treatment with a statin for up to 12 weeks significantly improved EF (increase in EF domain score = 7.8; p = 0.04), and the clinical benefits of statins on EF were observed as early as treatment week 6 (140). Nightly treatment with a PDE5 inhibitor has also been shown to be effective in enhancing EF in patients with arteriogenic ED (141); many patients with inadequate responses to PDE5 inhibitors after prostatectomy have arterial insufficiency (142,143). Certain studies have suggested that enhanced control of cardiovascular risk factors and/or statin treatment may increase the efficacy of PDE5 inhibitors (vs. placebo + PDE5 inhibitors) (144,145).

#### Prostate cancer

All three PDE5 inhibitors can improve EF in patients after nerve-sparing radical prostatectomy and other procedures for prostate cancer (83,146–153). Bilateral nerve-sparing radical retropubic prostatectomy, with preservation of neurovascular bundles, is important for adequate responses to sildenafil; other factors associated with favourable treatment outcomes include age ≤ 65 years, better preoperative EF and a > 6-month interval after surgery, whereas androgen deprivation therapy may adversely affect responses to PDE5 inhibitors (148,149).

Some patients may benefit from nightly treatment with sildenafil after prostate surgery (150,151). In one study, men with nerve-sparing prostatectomy were randomised to nightly sildenafil (50 or 100 mg) for up to 36 weeks (150). Among men randomised to active treatment, 27% experienced spontaneous erections compared with 4% receiving placebo (p = 0.02). In a long-term study of 43 men with prostate cancer who received one radioactive seed implantation, 31 (72%) continued to have satisfactory responses to sildenafil at treatment year 3, with both men and their spouses having favourable treatment satisfaction (Erectile Dysfunction Inventory of Treatment Satisfaction scores > 70) (152). Among the 12 men who discontinued treatment, five (42%) cited suboptimal efficacy, six (50%) a return of erections adequate for vaginal penetration and one (8%) death of spouse. Residual nocturnal penile tumescence and rigidity may predict more successful PDE5 inhibitor therapy after prostate and other urological procedures (153).

#### Major depressive disorder

There is a potential bidirectional relationship between ED and depression. Treatment with certain antidepressants may be associated with ED and other forms of sexual dysfunction (see Table 1). Treatment with PDE5 inhibitors can enhance sexual function in men with ED secondary to, or in the presence of, depression or antidepressants, including medications affecting serotonergic pathways (154–163). Sexual dysfunction in the setting of severe or treatment-refractory depression warrants referral to a specialist.

### Help the patient and partner identify alternative (non–PDE5-based) satisfactory therapy

Given the subjective nature of sex and other individual relationship issues, many couples will benefit from using different treatments for ED. Recent evidence suggests that patients who tried all three PDE5 inhibitors had improved outcomes, including long-term treatment adherence (164). Other studies have suggested that some patients will experience favourable responses to one PDE5 inhibitor after suboptimal responses to another (165).

On the other hand, no treatment (including first-line PDE5 inhibition) is uniformly well suited to all patient and partner needs. Some couples will experience superior outcomes with other, second- or third-line forms of therapy, including ICIT (166–173) or alprostadil suppositories, sublingual apomorphine (in non-US markets), vacuum erection/constriction devices (174–176) and/or surgical implantation of penile prostheses or other devices (177–181), either alone or combined/alternating with PDE5 inhibitors. In some cases, clinically occult sexual arousal disorders can complicate treatment with PDE5 inhibitors, which require sexual arousal (94). On the other hand, the effectiveness of ICIT, vacuum erection devices and penile prostheses are typically not adversely affected by arousal difficulties in the ED patient (94).

### Potential limitations

Limitations of our analysis include the possibility of publication bias, in which studies showing less pronounced benefits of PDE5 inhibitors on treatment outcomes might not have been published, leading us to overestimate the salutary effects of these treatments. Most of the studies we evaluated involved relatively short treatment intervals (< 1 year); only data on heterosexual couples were assessed; and we did not specifically evaluate factors affecting treatment outcomes in patients with diabetic ED or potential predictors of response to each PDE5 inhibitor (182–191). We did not include agents that are investigational or approved in other parts of the world. Finally, our mnemonic EPOCH, while evidence-based overall, has not been validated as an effective treatment strategy through prospective, randomised, controlled studies or other forms of clinical experience.

## Conclusions

Erectile dysfunction is a major health issue affecting many adult men, impairing emotional well-being, satisfaction and overall QOL. Most men do not discuss the problem with their physicians and/or receive treatment. If they do, many patients and their partners abandon therapy. PDE5 inhibitors have become first-line non-invasive treatments for ED. The literature demonstrates that successful treatment of this condition frequently improves QOL, relationship metrics and overall sexual satisfaction. In an attempt to optimise long-term outcomes with PDE5 inhibitors, including treatment satisfaction and long-term adherence, physicians need to educate patients and their partners. Key elements towards optimising ED treatments include: (i) evaluating and educating patients and partners to ensure realistic expectations of therapy; (ii) prescribing a treatment well suited to the couple’s lifestyle needs and other preferences; (iii) optimising treatment by scheduling follow-up visits with the patient to ‘fine-tune’ dosages and revisit key educational messages; (iv) controlling comorbidities via lifestyle counselling, medications and/or referrals and (v) helping patients and their partners to address their health and psychosocial needs.
